# Genomic Investigation of a *Mycobacterium tuberculosis* Outbreak Involving Prison and Community Cases in Florida, United States

**DOI:** 10.4269/ajtmh.17-0700

**Published:** 2018-07-09

**Authors:** Marie Nancy Séraphin, Xavier Didelot, David J. Nolan, Justin R. May, Md Siddiqur Rahman Khan, Ellen R. Murray, Marco Salemi, J. Glenn Morris, Michael Lauzardo

**Affiliations:** 1 Division of Infectious Diseases and Global Medicine, College of Medicine, University of Florida, Gainesville, Florida;; 2 Emerging Pathogens Institute, University of Florida, Gainesville, Florida;; 3 Department of Infectious Disease Epidemiology, Imperial College London, London, United Kingdom;; 4 Department of Pathology, Immunology and Laboratory Medicine, University of Florida, Gainesville, Florida;; 5 Bioinfoexperts, LLC, Thibodaux, Louisiana

## Abstract

We used whole-genome sequencing to investigate a tuberculosis outbreak involving U.S.-born persons in the prison system and both U.S.- and foreign-born persons in the community in Florida over a 7-year period (2009–2015). Genotyping by spacer oligonucleotide typing and 24-locus mycobacterial interspersed repetitive unit-variable number tandem repeat suggested that the outbreak might be clonal in origin. However, contact tracing could not link the two populations. Through a multidisciplinary approach, we showed that the cluster involved distinct bacterial transmission networks segregated by country of birth. The source strain is of foreign origin and circulated in the local Florida community for more than 20 years before introduction into the prison system. We also identified novel transmission links involving foreign and U.S.-born cases not discovered during contact investigation. Our data highlight the potential for spread of strains originating from outside the United States into U.S. “high-risk” populations, such as prisoners, with subsequent movement back to the general community.

## INTRODUCTION

In the United States, universal and systematic genotyping of *Mycobacterium tuberculosis* cases by spacer oligonucleotide typing (spoligotyping) and 24-locus mycobacterial interspersed repetitive unit–variable number tandem repeat (MIRU-VNTR) typing through the U.S. National Tuberculosis (TB) Genotyping Service has been instrumental in the understanding of the molecular epidemiology of TB.^1,2^ The service also monitors progress toward elimination by quantifying recent TB transmission rates.^3,4^ Genotyping has greatly improved TB prevention and control efforts in the United States by facilitating the prompt identification and treatment of latently infected cases.^1,5^ However, the system is not without limitations. A well-functioning TB program will see their TB genotype clusters grow over time as new TB cases infected with endemic strains are diagnosed.^6,7^ In addition, immigration from high-incidence countries will also contribute to the expansion of *M. tuberculosis* genotype clusters when persons settle in limited geographical regions within the adopted country.^7^ In cases where a genotype cluster cannot be resolved by contact tracing, whole-genome sequencing (WGS) can be implemented selectively as a secondary genotyping step to describe TB clusters at a higher resolution.^8,9^ Nevertheless, although WGS provides unprecedented resolution to TB outbreak investigation, traditional epidemiological investigation of contacts is still crucial to define transmission links.^8,9^ Retrospective evaluation of genotype clusters may benefit from contemporary methods that combine genomic data with mathematical models to reconstruct TB outbreaks and provide TB control programs with actionable information, such as the probability of person-to-person transmission within TB clusters.^10,11^

In this study, we used WGS, phylogenetics, and transmission modeling to investigate FL0117, a decade-old TB genotype cluster involving U.S.- and foreign-born persons in Florida, United States. Most of the U.S.-born cases were diagnosed while incarcerated or in the community following exposure to the prison population. The rest of the cases were foreign-born persons without a history of incarceration. The goal of this investigation was to define recent transmission clusters, estimate the timing and directionality of transmission, and assess possible origins for the FL0117 outbreak strain.

## METHODS

### Cluster description.

In March 2009, the Florida Department of Health (FDOH) received notice of an infectious case of TB at a Florida County Prison (Facility C), which triggered an outbreak investigation. Twenty-seven more cases were detected among the prison population between March 2009 and October 2013. Genotyping by spoligotyping and 24-locus MIRU-VNTR on culture-confirmed cases showed that they all shared identical genotype profile. The cases were assigned to the genotype cluster FL0117. A review of the Florida TB Genotyping Information Management System,^5^ identified 44 more cases with the same genotype profile reported in the community between 2003 and 2015.

### Epidemiologic investigation.

State TB control staff members collected extensive clinical, sociodemographic, and risk factor data on each reported TB case to guide case management and control efforts as part of the National TB Surveillance System.^12^ A Report of Verified Case of TB was available for all FL0117 cases. Disease investigation staff reviewed past prison outbreak reports to identify a source for the prison cases and conducted contact tracing in the community in an attempt to link the two populations.

### Genomic investigation.

Full methods for culturing, sequencing, and phylogenetic reconstruction are available in the Supplemental Information. In summary, all viable isolates associated with the FL0117 cluster were prepared for 2 × 250 paired end sequencing on the Illumina MiSeq system (Illumina, Inc., San Diego, CA), using Nextera XT library construction kits following manufacturers’ instructions. After quality trimming with Trimmomatic,^13^ we assembled the reads to the reference genome, CDC1551 (accession no. AE000516) and identified single-nucleotide polymorphisms (SNPs). We identified high-quality SNPs (hqSNPs) with adequate coverage (> 20 reads on average per site) across all sequenced isolates and constructed a FASTA file of hqSNPs with the date of isolation for each sample in months since 2003, using the year 2003 as point of reference for when the FL0117 genotype was first reported in Florida. We tested the temporal signal of the genomic data by maximum likelihood (ML) mapping and regression methods using the software packages IQTree v1.4.2 and TempEst v1.5, respectively.^14,15^

### Phylogenetic reconstruction and inference of person-to-person transmission.

We evaluated the evolutionary relationships between the genomes using a minimum spanning tree (MST).^16^ We compared the pairwise genetic distance within and between prison and community cases, foreign-born, and U.S.-born cases using the software program MEGA7.^17^ In a preliminary analysis, we used an SNP cutoff of ≤ 5 to define recent transmission between any two cases.^9^ We used the software package BEAST v 1.8.3 to infer the timescale of the outbreak, using the Haseqawa-Kishino-Yano nucleotide substitution model and a predefined strict molecular clock prior of 0.5 mutation per genome per year as previously estimated.^18,19^ The molecular clock rate was not estimated because of the limited temporal signal detected in TempEst analysis (Supplemental Figure 3). A maximum clade credibility (MCC) tree from the posterior distribution of trees was used for subsequent transmission analyses using the TransPhylo package in R version 3.3.2.^10,20^ Because TB cases with the FL0117 genotype were still being reported at the time these analyses were performed, we inferred the transmission probabilities under the “ongoing outbreak scenario.”^10^

### Ethical considerations.

The data were collected as part of public health practice and, thus, participants were not consented. The Institutional Review Boards of the University of Florida and the FDOH approved the use of the data for this study, and all data were de-identified centrally at FDOH before transfer to our group for analyses.

## RESULTS

### Clinical and sociodemographic characteristics of the cases.

**Figure 1.**
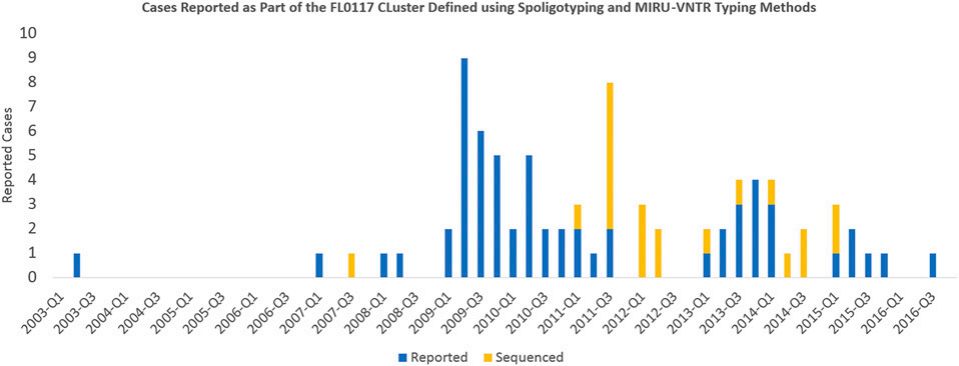
Epidemic curve of the spoligotyping and mycobacterial interspersed repetitive unit–variable number tandem repeat (MIRU-VNTR) defined *Mycobacterium tuberculosis* cluster, FL0117. Orange bars indicate sequenced isolates as a proportion of all cases within the cluster reported in that quarter. This figure appears in color at www.ajtmh.org.

The FL0117 genotype was first reported in May 2003, and as of 2015, 81 other cases were reported. The first case that initiated the outbreak in the prison was diagnosed in March 2009. Cases were continuously reported throughout the rest of 2009, first peaking early in 2009; a second peak occurred by late 2011 (Figure 1). Of the 82 total cases reported, 51 (62.2%) were born in the United States. The mean age at diagnosis for the sample was 41.8 (standard deviation [SD] = 16.5) years, with no significant difference between U.S.- and foreign-born cases (42.2, SD = 15.4 years versus 41.1, SD = 18.3 years; *P* = 0.2746) (Table 1). The majority of the cases were male (67.1%) and Black/African American (73.2%). Close to 88% had pulmonary TB and half were sputum smear positive. At the time of diagnosis, 30 (36.6%) were incarcerated, 23 (28.1%) were co-infected with the human immunodeficiency virus (HIV) 14 (17.1%) had a history of injection and non-injection drug use, seven (8.5%) abused alcohol, and four (4.9%) were homeless in the year before diagnosis. Among the foreign-born cases, 54.8% (17/31) had lived in the United States for 5 years or more and 19.4% (6/31) reported living outside of the United States. There was no significant difference by country of birth in HIV status, sputum smear status, TB disease site, or treatment outcome. However, compared with the U.S.-born cases, those born outside of the United States were predominantly female and Black/African American and less likely to use illicit drugs (Table 1).

**Table 1:** Characteristics of the Cases Involved in the Outbreak by Birth Origin, Florida, 2009–2016

Characteristics	Sample *N* (%)	U.S.-born *n* (%)	Foreign-born *n* (%)
Total sample	82	51	31
Age (SD)*	41.8 (16.5)	42.2 (15.4)	41.1 (18.3)
Gender			
Male	55 (67.1)	44 (86.3)	11 (35.5)
Females	27 (32.9)	7 (13.7)	20 (64.5)
Race/ethnicity			
Black	60 (73.2)	31 (60.8)	29 (93.6)
White	18 (22.0)	17 (33.3)	1 (3.2)
Others	4 (4.8)	3 (7.8)	1 (3.2)
Tuberculosis type			
Pulmonary	72 (87.8)	46 (90.2)	26 (83.9)
Pleural	2 (2.4)	2 (3.9)	–
Other	8 (9.8)	3 (5.9)	5 (16.1)
Sputum smear status†			
Negative	36 (50.00)	24 (51.1)	12 (48.0)
Positive	36 (50.00)	23 (48.9)	13 (52.0)
Treatment outcome			
Completed	72 (87.8)	44 (86.3)	28 (90.3)
Refused	1 (1.2)	1 (2.0)	–
Died	4 (4.9)	2 (3.9)	2 (6.5)
Others	5 (6.1)	1 (2.0)	1 (3.2)
Risk factor			
Diagnosed while incarcerated	30 (36.6)	30 (58.8)	–
Past year alcohol abuse	7 (8.5)	7 (13.7)	–
Past year drug use‡	14 (17.1)	13 (25.5)	1 (3.2)
Past year homelessness	4 (4.9)	4 (7.8)	–
HIV positive	23 (28.1)	15 (29.4)	8 (25.8)
Number of years in the United States§			
< 5 years	17 (54.8)	–	17 (54.8)
≥ 5 years	14 (45.2)	–	14 (45.2)
Live outside the United States	6 (19.4)	1 (2.0)	6 (19.4)

* Indicates mean age and standard deviation (SD).

† Among pulmonary cases.

‡ Includes injection and non-injection drug use.

§ Among foreign-born cases.

### Phylogenetic reconstruction and inference of recent transmission.

**Figure 2.**
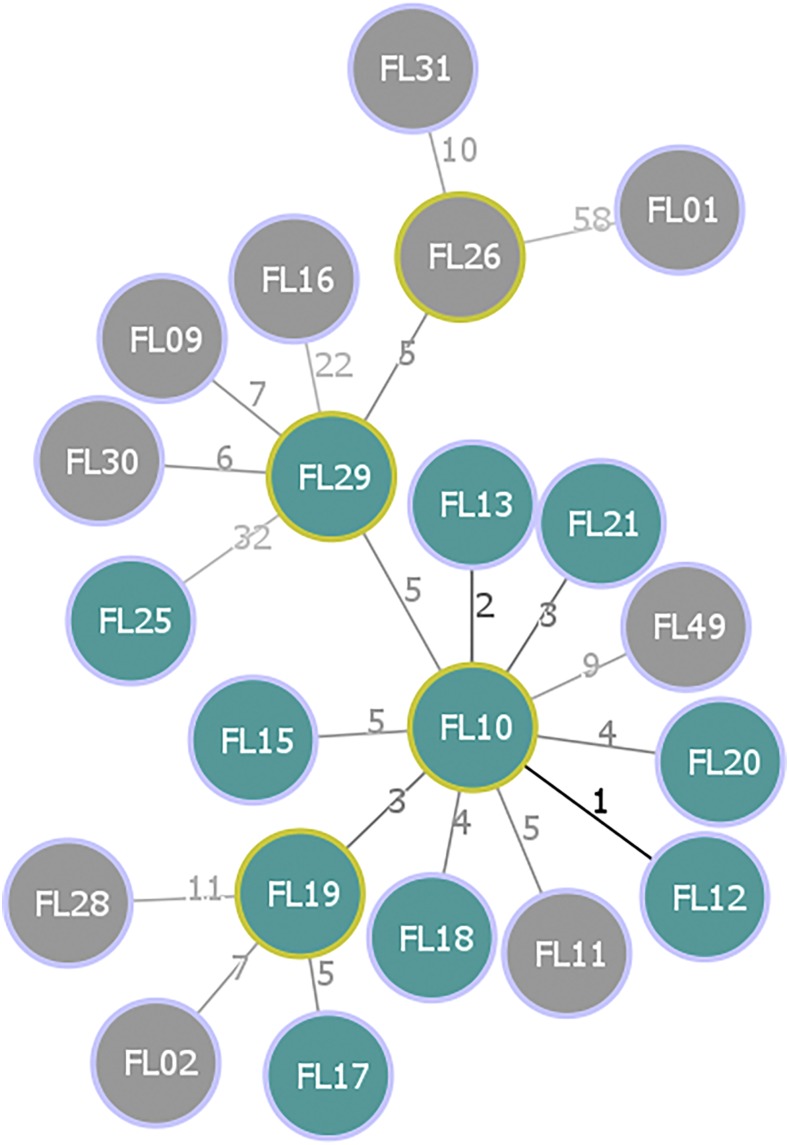
Phylogenetic reconstruction of the FL0117 cluster. Nodes represent each of the sequenced cases (*N* = 21). Gray identifies the foreign-born cases and cyan identifies the U.S.-born cases. Yellow outline identifies central nodes. The numbers on the branches represent single nucleotide variant between pairs of isolates. This figure appears in color at www.ajtmh.org.

**Figure 3.**
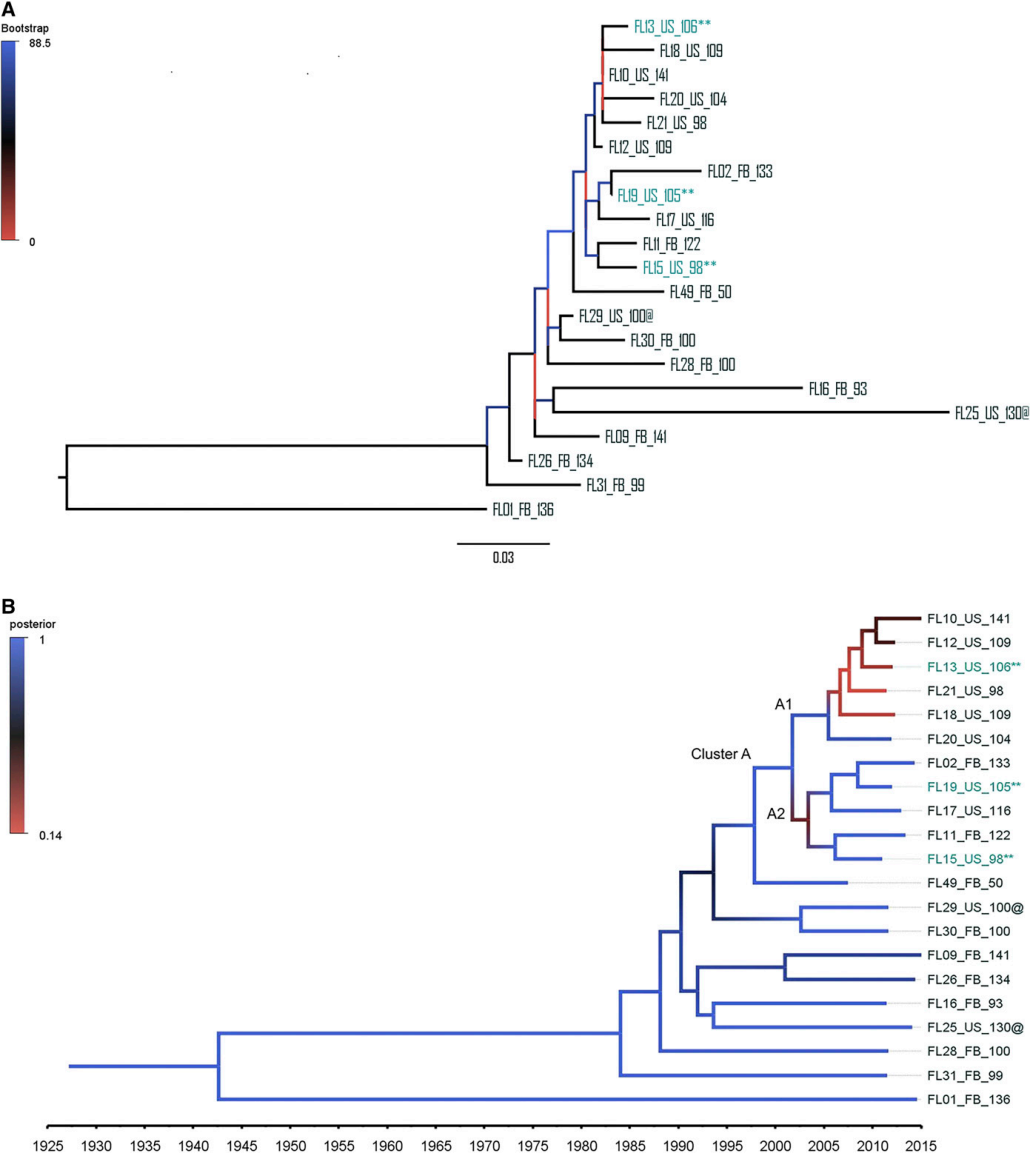
Estimated divergence time of the FL0117 cluster. (**A**) Midpoint rooted maximum likelihood phylogeny. (**B**) Timed-labeled maximum clade credibility tree. The two-letter birth origin abbreviation is identified in the taxon name: US—U.S.-born and FB—foreign-born. Tip label colors indicate location at diagnosis: navy—community cases and cyan—Prison cases. @ denotes pediatric cases. Branch color represents the node bootstrap values (**A**) and the posterior distribution of trees (**B**). This figure appears in color at www.ajtmh.org.

Frozen stock for 53 of the 82 isolates associated with the FL0117 cluster were located and subcultured, and 21 were sequenced, including one isolate from 2007. We had an average of 88×, (SD = 42.7×) coverage across the 21 genomes. Sequencing coverage and epidemiological information about individual isolates are presented in Supplemental Table 1. Sequenced cases were a representative spatial and temporal sample of the cluster (Supplemental Figure 1). Overall, 134 SNPs differentiated the 21 FL0117 genomes. Nevertheless, genetic relatedness varied within subpopulations. Based on mean pairwise distances, the strains from the prison population were less evolutionarily diverse than strains isolated in the community (6 versus 21 SNPs). U.S.-born cases were closely related (11 SNPs), whereas strains isolated among the foreign-born were more genetically diverse (26 SNPs). The star-shaped topology of the MST illustrating the evolutionary history between the cases (Figure 2) strongly suggested the presence of a super-spreader responsible for most of the U.S.-born cases. Using a cutoff of ≤ 5 SNPs,^9^ we defined one recent transmission cluster composed of 10/11 U.S.-born cases (the exception being FL25), including the three sequenced prison cases (FL13, FL15, and FL19), and 2/10 foreign-born cases (FL11 and FL26). Based on the observed genomic diversity, we evaluated the ML and Bayesian phylogenetic reconstructions testing the hypothesis that the FL0117 cases represented at least three distinct *M. tuberculosis* strain clusters that shared a recent common ancestor. The a priori likelihood mapping analysis (Supplemental Figure 2) confirmed that the SNP alignment would reliably resolve the evolutionary relationship. Both the ML and the MCC phylogenies supported the genomic clusters inferred from the SNP analyses. We estimated the divergence time for the strains to be about 1,942.6 (95% highest posterior density [HPD]: 1,926.9, 1,957.2). One major clade, termed Cluster A, could be observed from the ML (Figure 3A) and MCC phylogenies (Figure 3B). The estimated node age for Cluster A dates to 2,001.8 (95% HDP: 1,998.1, 2,005.4) and includes all the cases involved in the recent transmission cluster defined previously. We further divided Cluster A into Clade A1, composed of U.S.-born cases, and the mixed U.S.-born and foreign-born Clade A2. All other strains were isolated from foreign-born cases and were distantly related to each other, indicating that the FL0117 genotype circulated in a foreign country for a much longer period than in Florida, where accumulation of genetic diversity and varied transmission networks are represented by long branch lengths between foreign-born cases (Figure 3B).

### Origin of the prison outbreak.

The historical isolate, FL49, was recovered in 2007 from a foreign-born woman who immigrated to Florida 2 years before her diagnosis with cavitary TB. FL49 is closely related to Cluster A and more distantly related to the other foreign-born cases identified as part of the outbreak (12 versus 25 SNPs, respectively). FL49 is, therefore, the closest sequenced relative for the strain that caused the outbreak in the prison population. It is likely that the outbreak source strain circulated in the community at least 5 years before the first reported prison cases in 2009 (Figure 3B). At the time of her diagnosis, FL49 did not trigger a contact investigation as she was a recent arrival and genotyping was not yet systematically performed in Florida.

### Putative person-to-person transmission.

Compared with strains in Cluster A, the other strains were evolutionarily more diverse; we estimated two separate time-labeled phylogenies and used them as input into TransPhylo to establish putative transmission links. We extracted all pairwise probabilities of transmission, and all were close to zero for the foreign-born cases (Supplemental Table 2). For Cluster A, none of the transmission probabilities was inferred strongly (< 25%), thus supporting the hypothesis for unsampled transmission intermediates and limited direct person-to-person transmission between the sampled cases. The highest transmission probabilities inferred using TransPhylo reflected contact investigation findings by FDOH linking all U.S.-born cases to the Department of Correction (DOC), either as a former inmate (FL12) or contact to an inmate upon release to the community (FL18, FL17, FL20, and FL21). We estimated a transmission probability of 17.8% between FL10 and FL12; however, FL10 was not investigated by FDOH. No DOC connection was identified for the foreign-born cases during contact investigation, nor could they be linked to each other, except for the newborn (FL29) and her babysitter (FL30). However, both the ML and the time-labeled phylogenies (Figure 3) support a shared recent transmission history between the prison cases (FL19 and FL15) and two foreign-born cases in the community (FL11 and FL02). It is unknown whether the foreign-born cases had ever been incarcerated in Florida.

**Figure 4.**
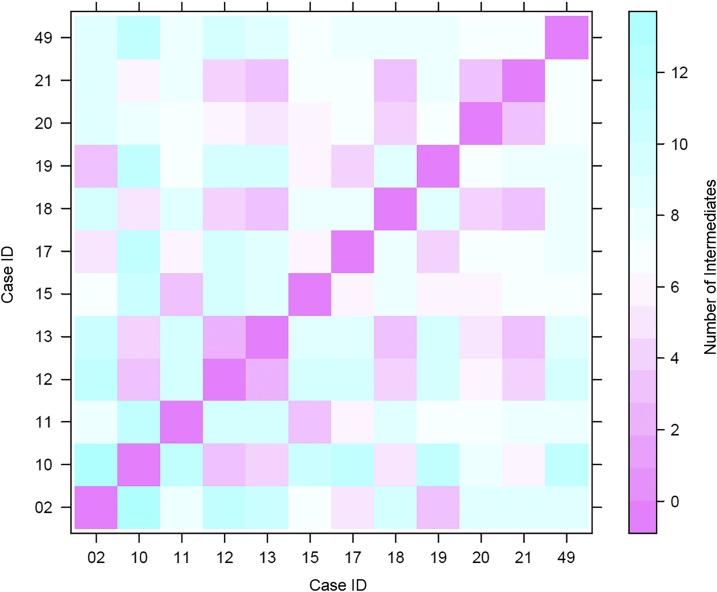
Average number of intermediates in the transmission chain between any two cases involved in the prison outbreak. The data are presented in a pairwise matrix with the color gradient from pink to cyan, indicating an increasing number of intermediates in the transmission chain from one case to another. This figure appears in color at www.ajtmh.org.

Because the outbreak was partially sampled, we used the TransPhylo output to infer the number of intermediate links in the transmission chains (Figure 4). On average, there were a minimum of two and a maximum of 14 intermediate links between any two cases in Cluster A. Five intermediates was the lowest computed links in the transmission chain between FL29 and FL30. The two cases are separated by a six-SNP difference, which is within the SNP limit for household transmission. However, the symmetric relationship between these two cases in the time-labeled phylogeny (Figure 3) suggests that someone else may have infected the two cases. All other pairs had a minimum of nine and maximum of 36 links between them. These observations support the hypothesis that little to no recent transmission occurred within the foreign-born population and cases were mainly due to delayed progression to active disease post-immigration. Indeed, beyond the confirmed epidemiological link between FL29 and FL30, no other connections could be identified for the other foreign-born cases involved in the outbreak.

### Strain introduction and transmission within the prison population.

We identified two novel transmission chains using the genomic data ([FL02–FL19, 7 SNPs] and [FL11–FL15, 6 SNPs]), which establish putative transmission between two foreign-born cases in the community and the prison population (Figure 3). The two foreign-born cases had lived in the United States for over a decade and reported high-risk behaviors (drug use and HIV infection) that could predispose them to TB infection and transmission. Although the foreign-born cases were likely a result of DOC to community transmission, they nevertheless establish social interactions between the two populations that would facilitate foreign-born to U.S.-born transmission in the community and subsequent introduction of the outbreak strain into the prison population. These epidemiologic links were not identified during conventional contact investigation.

**Figure 5.**
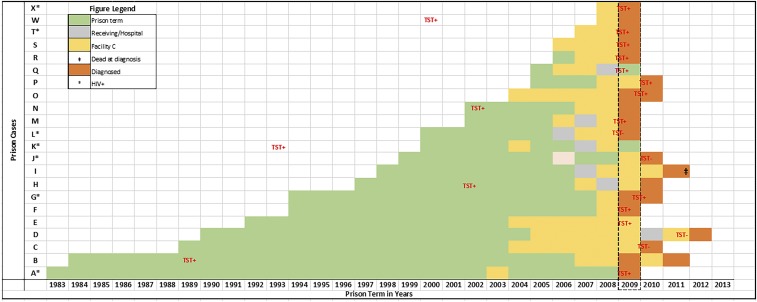
Reconstruction of inmates initial entry into the prison system and recorded movements before tuberculosis (TB) diagnosis. Year of entry into the prison system for each inmate (A–X) is given on the *x* axis. Each green box represents a year into their prison term with a change in color indicating movement from their current location to Facility C (yellow) or the main receiving/hospital facility (gray). The dashed box highlights the year the first TB cases were reported, 2009. TST = tuberculin skin test as an indication of TB infection status. Cases D (FL13) and B (FL15) were sequenced. This figure appears in color at www.ajtmh.org.

We had prison terms and movement information on 25 of the 30 inmates involved in the outbreak, including two of the three cases we sequenced. Historical TB skin test results were available for five inmates; two were positive before incarceration, including one who was HIV positive and the other three tested positive while incarcerated and several years before the first cases in 2009 (Figure 5). Inmates’ prison terms overlap for several years before diagnosis with TB disease between 2009 and 2010. More importantly, all inmates spent at least a year at correctional facility C before diagnosis. One inmate (I) died of causes unrelated to TB disease in 2011 and was diagnosed postmortem. He moved between several facilities and spent a year at the main receiving center/hospital before expiring at another facility in 2011. All but one inmate spent at least 1 year at Facility C before evaluation as a contact of an infectious TB case in 2009 and subsequent TB diagnosis. The frequent inmate movement with the influence of HIV coinfection contributed to the spread of the outbreak strain throughout DOC, exposing both inmates and staff. The movement data also highlight several missed opportunities to screen and treat latent infection in the prison population.

## DISCUSSION

Through a multidisciplinary approach, we showed that a putative single outbreak first identified using traditional genotyping markers actually involved distinct bacterial transmission networks segregated by country of birth. One cluster of genetically related strains encompassed all of the prison isolates sequenced and cases where recent contact with a released prisoner was reported, and could be subdivided into two smaller transmission clusters based on whole-genome phylogenetic analysis. The remaining cases, composed almost entirely of persons born outside the United States, involved multiple introduction of a strain endemic to a foreign country, with isolated transmission events to close household contacts in Florida. Our findings are consistent with reports from similarly low-incidence settings in the United Kingdom, Canada, Switzerland, and Australia, where it has been found that traditional genotyping overestimates local transmission.^9,21–23^ Within mixed native and foreign-born clusters in low-incidence settings, it has also been found that transmission is limited to the native-born population.^9,21,23^ Our data also highlight the increasing need to implement WGS and associated analytic tools routinely within TB control programs in low-incidence settings.^24,25^

United States State TB control programs use traditional genotyping routinely for TB outbreak investigation, with in-depth source identification and contact tracing on major genotype clusters.^26,27^ Whole-genome sequencing is only conducted occasionally on interesting clusters, such as the one we report here. However, in agreement with prior studies, our results illustrate the inferior discriminatory power of 24-locus MIRU-VNTR typing to distinguish between closely related *M. tuberculosis* clones, thus overestimating transmission events.^21,22,24^ There have also been instances, including in this study, where traditional genotyping coupled with contact investigation have been unable to establish recent transmission.^9^ Likely, WGS could be used prospectively to link cases to an outbreak before traditional epidemiological investigation, thus saving time and resources, especially given the limited yield of name-based contact tracing in high-risk, transient populations such as the homeless, prisoners, and drug users.^8,28^ In our study, FDOH discontinued contact investigation in 2014 when a link between the foreign-born population and the U.S.-born population could not be established. Using WGS and phylogenetics, we showed putative transmission from the prison population to the foreign-born population in the community. Had this type of analysis been performed near the time of sample collection and outbreak investigation, it would have established probable transmission links between cases and generated leads for contact tracing both in the prison and local jails in an attempt to link the two populations.

A limitation of our study is that we only sequenced a quarter (21/82) of the cases included in the FL0117 cluster. Some of the isolates could not be recovered, whereas others were not viable on subculture, limiting the final number of isolates available for sequencing (Supplemental Figure 1). However, in-depth analysis of the selected isolates showed that they were a representative spatial and temporal sample of the cluster (Supplements), confirming they would be able to accurately reconstruct the phylogenetic history of the cluster. Nevertheless, the bacterial diversity is larger than represented in our sampling, which would add more branches to the phylogenetic reconstruction presented in this study. However, we believe that the low sampling proportion likely led to an underestimation rather than an overestimation of the true genetic distance between epidemiologically unrelated cases in the FL0117 cluster, that is, foreign-born cases diagnosed in the community. For example, we have observed that one case (FL01) found to be unrelated to the cluster by contact tracing was separated from all other genomes by at least 50 SNPs. Indeed, FL01 had arrived in Florida less than 3 months before diagnosis with TB disease in 2014, toward the end of the prison outbreak. In addition, in computing transmission events, we used validated methods suited for partially sampled outbreaks.^10^ In this study, we used an SNP cutoff of ≤ 5 to define recent transmission and considered cases with larger genetic distances as delayed progression to active disease.^9^ This decision was supported by the contact investigation data. Nevertheless, the strict SNP cutoff used here negates possible within-host evolution and potential distortion of the transmission events as a result.^9,29,30^ However, the minimum SNP cutoff to define recent transmission remains a source of contention in *M. tuberculosis* genomics.^31^ Unfortunately, as we did not sequence cases unrelated to this cluster, we are not sure what the expected SNP difference would be between cases with discordant genotypes in the study region.^9,22^ These data would have also allowed us to account for within-host diversity when defining a SNP cutoff for recent transmission.^9^

Our study results have significant implications for TB control in the United States. As of 2016, 6.7 million persons were under the supervision of the U.S. Correctional System, either in jail, prison, or on parole.^32^ In Florida, 101,424 persons were incarcerated in the prison system during the period covering this study.^33^ Compared with the general adult population, inmates are at increased risk for infectious diseases, including *M. tuberculosis*, because of overcrowding and lack of infection control in the correctional facilities.^34,35^ In addition, frequent and abrupt inmate transfers and releases hinder proper treatment and follow-up while facilitating the spread of infectious pathogens.^34^ Likely, an important source of pathogen introduction into the prison system is the transient jail population that spends short periods in correctional facilities (hours to days).^34^ The jail population represents a bridge population between the community and the prison system because of the frequent re-entry and short stay, which does not allow for effective TB control measures, such as latent TB infection screening and treatment, to be implemented.^34^ We believe the jail population played a significant role in the introduction of the outbreak strain in the prison system in Florida. We have shown using WGS and associated analytical techniques that traditional genotyping methods coupled with contact tracing is insufficient for TB outbreak investigation. Mycobacterial interspersed repetitive unit-variable number tandem repeat and spoligotyping markers target less than 1% of the *M. tuberculosis* genome and are susceptible to convergent evolution or the chance occurrence of the same or similar genotyping pattern in phylogenetically unrelated cases.^36^ Thus, there is a clear need to implement WGS as a primary typing method within control programs in low TB-incidence countries.^24^ One of the strengths of our analyses is the extensive contact investigation data collected independently by the state, which allowed us to validate our genomic and TransPhylo analyses. The collection of such data is nevertheless time consuming and resource intensive. Prior studies and our own data, nevertheless, show that WGS correlates very well with contact investigation data.^9,37^ Using WGS and associated analyses as a first-line typing system to guide contact investigation have the potential to save time and resources while overcoming the limits of name-based contact tracing for TB outbreak investigation.

Whole-genome sequencing is quickly becoming the method of choice to study the evolutionary history of monomorphic pathogens such as *M. tuberculosis*.^38^ The integration of TB genomic data and mathematical models promises to revolutionize outbreak investigation by tracking transmission events in near real-time, thus narrowing down the pool of potential contacts to investigate.^10,11^ By integrating WGS, and phylogenetic and transmission modeling, we show that sequencing a representative sample of a TB cluster is enough to delineate an extensive TB cluster, infer the timing of transmission events, and inform public health decisions on resource allocations for contact tracing.

## Supplementary Material

Supplemental information, tables, and figures
