# Supplementary material for: Genomic Investigation of a Mycobacterium tuberculosis Outbreak Involving Prison and Community Cases in Florida, United States
Source: Am J Trop Med Hyg. 2018 Jul 9;99(4):867–74. doi: 10.4269/ajtmh.17-0700 (PMC6159577; doi:10.4269/ajtmh.17-0700)
Supplement: Supplementary file 1 [file tpmd170700.SD1.pdf]

Supplemental Methods

Strain Growth and DNA isolation

From March to July 2015, the Florida Department of Health Bureau of Public Health Laboratories (BPL) subcultured frozen isolates associated with the FL0117 cluster and shipping them to our group for sequencing. Some isolates could not be located and some were no longer viable. The isolates were grown on Middlebrook 7H11 agar plates, suspended in Middlebrook 7H9 broth, and shipped overnight to our lab. Upon arrival, they were transferred on to Middlebrook 7H11 plates and grown for six weeks to produce enough biomass for genomic DNA (gDNA) extraction using the CTAB method(1). DNA concentrations were measured using an Invitrogen Qubit 2.0, and quality assessment was done on agarose gels and the Agilent TapeStation 2200 system. Isolates with high molecular weight DNA and low levels of degradation or smearing on gels were selected. Sequencing libraries were prepared using Nextera XT library construction kits following manufacturers' instructions for bead-based normalization, except for modifying the number of PCR cycles from 12 to 15 to increase amplification of the libraries. 12-15 libraries were sequenced in each 2x250 paired-end sequencing run on the Illumina MiSeq system (Illumina, Inc., San Diego, CA). Overall, the primary isolates were subcultured two times, once by BPL and once by us. In cases we needed to repeat the extraction on a degraded sample, additional subcultures were done using the original BPL stock. **Table S1** shows isolation dates as well as the sequencing coverage for each of the sequenced isolates.

Genome Reconstruction and Variant Calling

Short, paired-end reads were quality trimmed with Trimmomatic and independently aligned to the *M. tuberculosis* reference genome, CDC1551 (accession no. AE000516) using Bowtie2(2). Variants were called in relation to the reference genome using FreeBayes (3) and filtered to remove those with highly clustered SNPs (SNPs within 3 nucleotides of another, with

supporting base quality less than 20, a read depth less than five or alternate allele frequency of 75%. We also filtered out variants identified in PE/PPE/PE_PGRS genes as well as transposons (IS-elements) and short repetitive elements (MIRU and CRISPR loci), using a custom bed-file. We identified 134 variable positions that differentiated the 21 genomes using MEGA7(4)and extracted those positions to a FASTA file. We labeled the FASTA file of these SNPs with the date of isolation in months for each sample in the form of “months since 2003”, using as point of reference the year (2003) the FL0117 genotype was first reported in Florida.

Data Analyses

Phylogenetic Analyses

We tested the temporal signal of the genomic data by maximum likelihood mapping and regression methods using the programs IQTree v1.4.2(5) and TempEst v1.5(6), respectively. We evaluated the evolutionary relationship between the genomes using the Minimum Spanning Tree (MST)(7). We compared the pairwise genetic distance within and between prison and community cases, Foreign and U.S.-born cases (4). We used BEAST v 1.8.3(8) to calibrate a strict molecular clock and infer the timescale of the outbreak, using the HKY(9) model and a strong clock prior (9.17×10^{-9})(10). We generated a posterior distribution of trees by running 1×10^{8} Markov Chain Monte Carlo (MCMC) generations, sampling every 1,000 steps. We used Tracer v1.5 to analyze the output and assess mixing, using an effective sample size (ESS) value above 200 as the cutoff value for proper mixing of the Markov chain. A maximum clade credibility (MCC) tree from the posterior distribution of trees was used for subsequent transmission analyses using the TransPhylo algorithm in R version 3.3.2.(11).

Inference of Person-to-Person Transmission

We used TransPhylo available at (https://github.com/xavierdidelot/TransPhylo) to infer a transmission tree using the timed-labelled phylogeny produced in BEAST. TransPhylo is described in detail here(11,12). In brief, the model uses a stochastic branching process to estimate person-to-person transmission accounting for within-host variation and partial sampling of the outbreak cases. The algorithm has previously be used to infer timing of person-to-person transmission, which allowed public health officials to differentiate between delayed progression and recent transmission to declare a TB outbreak. We used TransPhylo to investigate the extent of direct transmission in our study by computing the probability of transmission between pairs of cases. Given the epidemiology of our outbreak, we hypothesized that direct transmission would be limited to the U.S.-born population. Since TB cases with the FL0117 genotype were being reported as of these analyses, we inferred the transmission probabilities under the "ongoing outbreak scenario"(11).

Comparison between sequenced and reported cases

We recovered less than half of the FL0117 cluster isolates from BPL. In addition, we were not able to successfully subculture some of the isolates we received. As one of our goals was to evaluate how cases are related, we wanted to assure that sequenced isolates were representative of the putative outbreak. The clinical and socio-demographic characteristics of sequenced and non-sequenced FL0117 strains were compared using chi-square test or Fisher's exact test as appropriate. We used ArcGIS v10.1 (ESRI, Redlands, CA) to geocode all FL0117 cases to the centroid of the reported patient residential zip code at the time of diagnosis. The geographical coordinates were obtained from a polygon of the five-digit Florida ZIP Code Areas downloaded from the Florida Geographic Data Library (FGDL) and current as of 2012(13). Unweighted spatial means were computed with the corresponding distributional ellipse and

deviational ellipse within one standard deviation of the spatial mean for both reported and sequenced isolates in order to evaluate that sequenced isolates were similar in spatial distribution to the reported cases. We were interested in assessing that the spatial mean for the sequenced isolates were within one standard deviation of the spatial mean for the reported cases, despite obvious deviation in the two mean centers. The retrospective space-time permutation model in SaTScan v9.4 was used to compare the spatiotemporal centroids of the sequenced and reported cases. The sequenced isolates did not cluster in space and time, indicating that sequenced cases were representative of the reported cases throughout the state.

Supplemental References

1. van Soolingen D, Hermans PW, de Haas PE, Soll DR, van Embden JD. Occurrence and stability of insertion sequences in Mycobacterium tuberculosis complex strains: evaluation of an insertion sequence-dependent DNA polymorphism as a tool in the epidemiology of tuberculosis. J Clin Microbiol. 1991 Nov;29(11):2578–86.

2. Langmead B, Trapnell C, Pop M, Salzberg SL. Ultrafast and memory-efficient alignment of short DNA sequences to the human genome. Genome Biol. 2009;10(3):R25.

3. Garrison E, Marth G. Haplotype-based variant detection from short-read sequencing. ArXiv12073907 Q-Bio [Internet]. 2012 Jul 17 [cited 2016 Apr 26]; Available from: http://arxiv.org/abs/1207.3907

4. Kumar S, Stecher G, Tamura K. MEGA7: Molecular Evolutionary Genetics Analysis version 7.0 for bigger datasets. Mol Biol Evol. 2016 Mar 22;msw054.

5. Nguyen L-T, Schmidt HA, von Haeseler A, Minh BQ. IQ-TREE: a fast and effective stochastic algorithm for estimating maximum-likelihood phylogenies. Mol Biol Evol. 2015 Jan;32(1):268–74.

6. Rambaut A, Lam TT, Max Carvalho L, Pybus OG. Exploring the temporal structure of heterochronous sequences using TempEst (formerly Path-O-Gen). Virus Evol [Internet]. 2016 Jan 1 [cited 2017 Mar 29];2(1). Available from: https://academic.oup.com/ve/article/2/1/vew007/1753488/Exploring-the-temporal-structure-of-heterochronous

7. Francisco AP, Vaz C, Monteiro PT, Melo-Cristino J, Ramirez M, Carriço JA. PHYLOViZ: phylogenetic inference and data visualization for sequence based typing methods. BMC Bioinformatics. 2012;13:87.

8. Drummond AJ, Suchard MA, Xie D, Rambaut A. Bayesian phylogenetics with BEAUti and the BEAST 1.7. Mol Biol Evol. 2012 Aug;29(8):1969–73.

9. Hasegawa M, Kishino H, Yano T. Dating of the human-ape splitting by a molecular clock of mitochondrial DNA. J Mol Evol. 1985;22(2):160–74.

10. Ford CB, Shah RR, Maeda MK, Gagneux S, Murray MB, Cohen T, et al. Mycobacterium tuberculosis mutation rate estimates from different lineages predict substantial differences in the emergence of drug-resistant tuberculosis. Nat Genet. 2013 Jul;45(7):784–90.

11. Didelot X, Fraser C, Gardy J, Colijn C. Genomic Infectious Disease Epidemiology in Partially Sampled and Ongoing Outbreaks. Mol Biol Evol. 2017 Apr 1;34(4):997–1007.

12. Didelot X, Gardy J, Colijn C. Bayesian inference of infectious disease transmission from whole-genome sequence data. Mol Biol Evol. 2014 Jul;31(7):1869–79.

13. University of Florida. Zip Code Areas (Five-Digit) in Florida - 2012 (FGDC) / U.S. ZIP Code Areas (Five-Digit)(ISO) [Internet]. [cited 2015 Apr 19]. Available from: http://www.fgdl.org/metadataexplorer/explorer.jsp

Supplemental Table 1. Isolate Description and Mapped read depth Information

Isolate	Year of Isolation	Mean Read Depth (expressed in X)	Gender	Age	Country Origin	Residence at Diagnosis	Sputum Smear Status
FL01_136	2014	24	Male	82	Vietnam	Community	Positive
FL02_133	2014	22	Male	59	Haiti	Community	Negative
FL09_141	2015	36	Male	63	Haiti	Community	Negative
FL10_141	2015	55	Male	34	U.S.	Community	Negative
FL11_122	2013	55	Male	42	Haiti	Community	Positive
FL12_109	2012	22	Male	48	Haiti	Community	Positive
FL13_106	2012	19	Male	50	U.S	Correction	Positive
FL15_98	2011	98	Male	49	U.S.	Correction	Positive
FL16_93	2011	88	Female	16	Haiti	Community	Positive
FL17_116	2013	117	Male	19	U.S.	Community	Negative
FL18_109	2012	129	Male	62	U.S.	Community	Negative
FL19_105	2012	134	Male	41	U.S.	Correction	Not done
FL20_104	2011	90	Male	35	U.S.	Community	Negative
FL21_98	2011	116	Male	48	U.S.	Community	Positive
FL25_130	2014	130	Female	4	U.S.[#]	Community	Not done
FL26_134	2014	108	Male	52	Grenada	Community	Not done
FL28_100	2011	125	Female	63	Haiti	Community	Positive
FL29_100	2011	141	Female	<1	U.S.[#]	Community	Not done
FL30_100	2011	126	Female	14	Haiti	Community	Positive
FL31_99	2011	113	Female	57	Haiti	Community	Not done
FL49_50	2007	100	Female	23	Haiti	Community	Positive

Notes: # pediatric cases born in the United States of at least one Haitian-born parent

Supplemental Table 2. Transmission Probabilities and Computed Intermediaries between Pairs of Cases.

Appendix Table S2 is a separate csv file of the pairwise transmission probabilities and intermediates from the TransPhylo analysis.

Supplemental Figure 1. Flow diagram of available isolates and selection of strains for sequencing. From 2003 to 2016, 82 TB cases were assigned the genotype ID FL0117 in Florida. Two isolates did not have culture available and five historical isolates from 2003 – 2008 were not officially linked to the outbreak. One of these isolates were sequenced (FL49) as part of the genomic investigation.

Supplemental Figure 2. Spatiotemporal Descriptive Statistics Comparing Outbreak and Sequenced Cases. Shifts in the mean centers of the two populations can be observed; however, there is complete overlap in dispersion and directionality. In addition, we did not observe a bias towards a specific geographical location or time, as sequenced isolates did not cluster spatiotemporally, unlike reported cases (black).

Supplemental Figure 3. Likelihood Mapping Analysis Testing the Phylogenetic Signal of the Sequence Alignment. Frequencies indicate the posterior probabilities for a set of possible unrooted phylogenies (n=525). The central triangle represents the posterior probability where all possible phylogenies are equally supported and are thus unresolved, while the rectangles represent areas where the data support conflicting tree topologies. The results indicate all sequence alignments included in our study will reliably reconstruct the outbreak phylogeny.

Supplemental Figure 4. Linear regression analysis of root-to-tip divergence versus sampling dates (in months) performed in TempEst

Note: Supplemental Figures 1, 2, 3 and 4 will be available online in final publication.
