# Supplementary material for: Genomic Investigation of a Mycobacterium tuberculosis Outbreak Involving Prison and Community Cases in Florida, United States
Source: Am J Trop Med Hyg. 2018 Jul 9;99(4):867–74. doi: 10.4269/ajtmh.17-0700 (PMC6159577; doi:10.4269/ajtmh.17-0700)
Supplement: Supplementary file 2 [file tpmd170700.SD2.pdf]

Transmission Probabilities for Cluster A

	FL02	FL10	FL11	FL12	FL13	FL15	FL17	FL18	FL19	FL20	FL21
FL02	0.0	0.0	0.0	0.0	0.0	0.0	1.0	0.0	7.8	0.0	0.0
FL10	0.0	0.0	0.0	9.8	1.6	0.0	0.0	0.0	0.0	0.0	0.0
FL11	0.0	0.0	0.0	0.0	0.0	7.2	0.4	0.0	0.0	0.2	0.0
FL12	0.0	17.8	0.0	0.0	7.2	0.0	0.0	0.2	0.0	0.0	0.6
FL13	0.0	2.8	0.0	22.6	0.0	0.0	0.0	0.2	0.0	0.0	3.8
FL15	0.2	0.0	9.2	0.0	0.0	0.0	2.4	0.0	0.8	0.4	0.0
FL17	1.8	0.0	1.2	0.0	0.0	1.4	0.0	0.0	3.8	0.0	0.0
FL18	0.0	0.0	0.0	0.2	4.8	0.0	0.0	0.0	0.0	4.0	12.6
FL19	8.2	0.0	0.6	0.0	0.0	0.0	3.2	0.0	0.0	0.0	0.0
FL20	0.0	0.0	0.2	0.0	0.6	1.0	0.0	12.4	0.0	0.0	3.0
FL21	0.0	1.0	0.0	3.0	17.8	0.0	0.0	4.0	0.0	1.6	0.0

Computed Intermediaries between Pairs of Cases for Cluster A

	FL02	FL10	FL11	FL12	FL13	FL15	FL17	FL18	FL19	FL20	FL21
FL02	0	14	8	12	11	7	5	10	3	9	10
FL10	14	0	12	3	4	11	12	7	12	8	5
FL11	8	12	0	11	10	3	6	8	6	8	9
FL12	12	3	11	0	2	10	10	5	11	6	4
FL13	11	4	10	2	0	9	9	4	10	5	3
FL15	7	11	3	10	9	0	5	7	5	7	8
FL17	5	12	6	10	9	5	0	8	4	7	8
FL18	10	7	8	5	4	7	8	0	8	4	3
FL19	3	12	6	11	10	5	4	8	0	8	9
FL20	9	8	8	6	5	7	7	4	8	0	4
FL21	10	5	9	4	3	8	8	3	9	4	0

Transmission Probabilities for Cases not in Cluster A

	FL01	FL09	FL16	FL25	Fl26	FL28	FL29	FL30	FL31
FL01	0.00	0.00	0.00	0.00	0.00	0.00	0.00	0.00	0.00
FL09	0.00	0.00	0.00	0.00	0.00	0.00	0.00	0.00	0.00
FL16	0.00	0.00	0.00	0.00	0.00	0.00	0.00	0.00	0.00
FL25	0.00	0.00	0.00	0.00	0.00	0.00	0.00	0.00	0.00
Fl26	0.00	0.00	0.00	0.00	0.00	0.00	0.00	0.00	0.00
FL28	0.00	0.00	0.00	0.00	0.00	0.00	0.00	0.00	0.00
FL29	0.00	0.00	0.00	0.00	0.00	0.00	0.20	0.00	0.00
FL30	0.00	0.00	0.00	0.00	0.00	1.00	0.00	0.00	0.00
FL31	0.00	0.00	0.00	0.00	0.00	0.00	0.00	0.00	0.00

Computed Intermediaries between Pairs of Cases not in Cluster A

	FL01	FL09	FL16	FL25	Fl26	FL28	FL29	FL30	FL31
FL01		36	35	36	36	33	34	34	33
FL09	36		13	15	9	16	15	15	19
FL16	35	13		11	13	14	13	13	17
FL25	36	15	11		14	16	15	14	19
Fl26	36	9	13	14		15	14	14	18
FL28	33	16	14	16	15		14	14	16
FL29	34	15	13	15	14	14		5	17
FL30	34	15	13	14	14	14	5		16
FL31	33	19	17	19	18	16	17	16	
