# Supplementary figures and images for: Genomic Investigation of a Mycobacterium tuberculosis Outbreak Involving Prison and Community Cases in Florida, United States
Source: Am J Trop Med Hyg. 2018 Jul 9;99(4):867–74. doi: 10.4269/ajtmh.17-0700 (PMC6159577; doi:10.4269/ajtmh.17-0700)

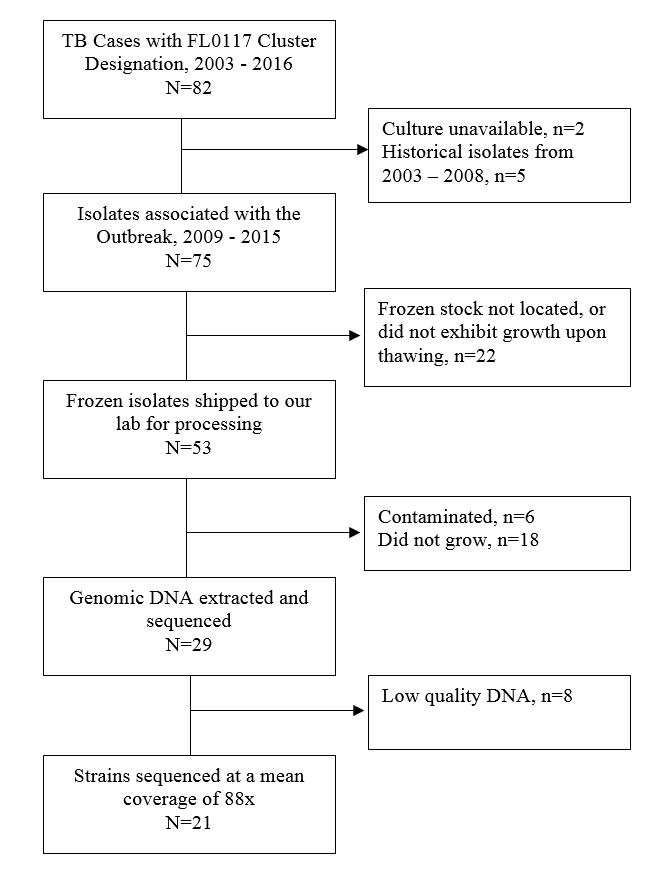

Supplement: Supplementary file 3 [file tpmd170700.SD3.tif]

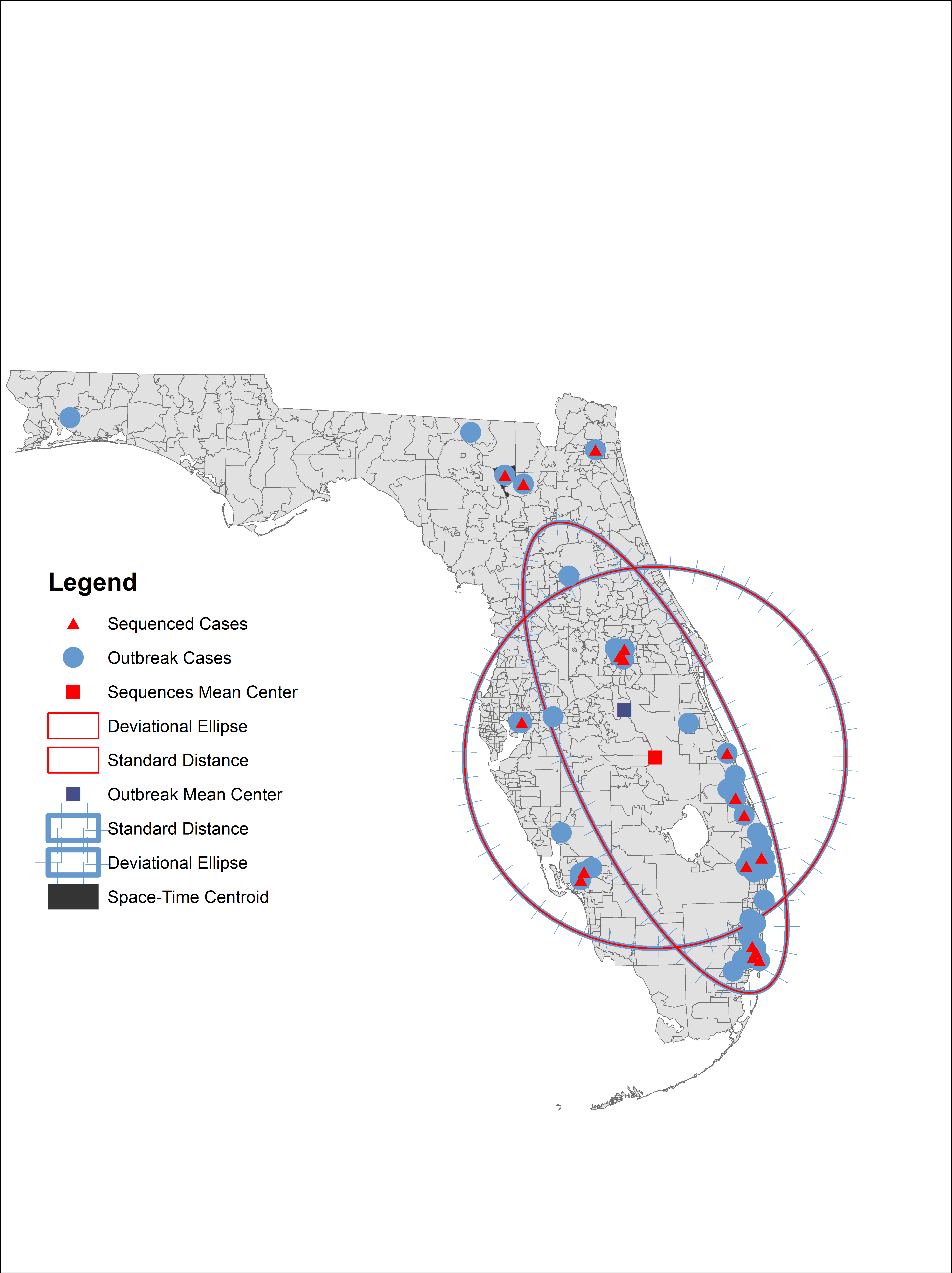

Supplement: Supplementary file 4 [file tpmd170700.SD4.tif]

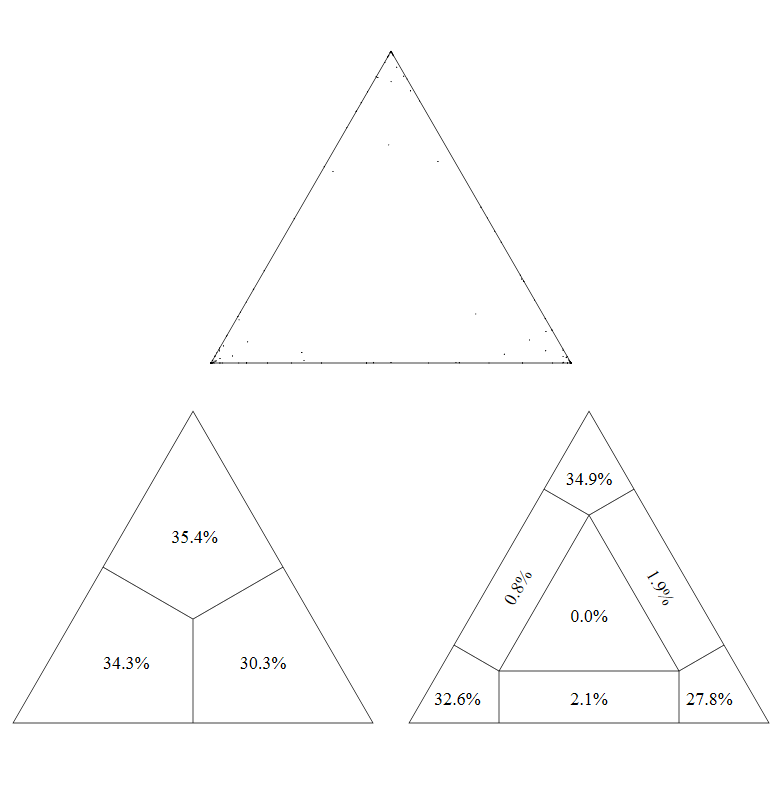

Supplement: Supplementary file 5 [file tpmd170700.SD5.tif]

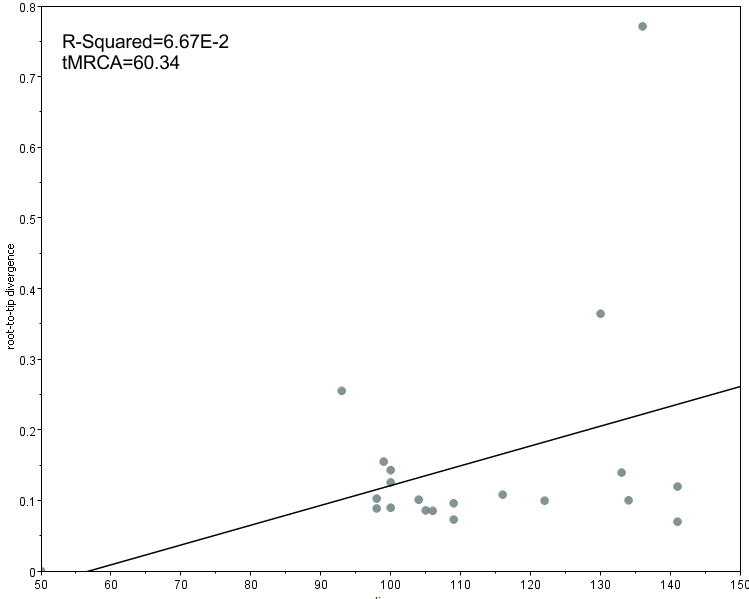

Supplement: Supplementary file 6 [file tpmd170700.SD6.tif]
